# Dental Management of a Child With Temtamy Syndrome: A Case Report

**DOI:** 10.7759/cureus.96193

**Published:** 2025-11-06

**Authors:** Khalid M AlHarbi, Mansour M Hakeem, Abdurrahman S Almutairy, Mohamed M Abouelnour, Lamis M Farghal

**Affiliations:** 1 Department of Pediatric Dentistry, Ohud Hospital, Madinah, SAU; 2 Department of Anesthesia, Ohud Hospital, Madinah, SAU

**Keywords:** c12orf57, case report, dental rehabilitation, micrognathia, temtamy syndrome

## Abstract

Temtamy syndrome is a rare genetic disorder that follows an autosomal recessive pattern. It is characterized by intellectual disability, developmental delays, and distinctive facial features. It may also affect the eyes, heart, and skeletal system. This case report details the dental treatment of a four-year-old male patient recently diagnosed with Temtamy syndrome. The patient complained of dental pain and presented with generalized developmental delay, intellectual disability, and hypotonia. Full dental rehabilitation under general anesthesia was planned due to behavior issues. Micrognathia and a limited mouth opening were among the challenges faced during the dental treatment. A limited mouth opening can be managed under general anesthesia, but if the child were to be treated under local anesthesia, this would further complicate the treatment. Therefore, the recommended treatment option when treating such patients is under general anesthesia to overcome these problems. This case report aims to discuss the oral features and dental management of children with Temtamy syndrome, which will aid fellow dentists in anticipating challenges faced in such cases, and further, understand the disease.

## Introduction

Temtamy syndrome is a rare autosomal recessive disorder. It was first noticed in 1991 by Temtamy et al. and later published in 1996 [1,2]. Pathogenic variants in chromosome 12 open reading frame 57 (*C12orf57*) were discovered in 2013 as the cause of the Temtamy syndrome [3,4]. The *C12orf57* gene is essential for the development of the corpus callosum [3,4]. Therefore, Temtamy syndrome involves abnormalities of the corpus callosum, either dysgenesis or agenesis [1-8].

Cases are presented with various neurological symptoms, MRI anomalies, and facial features. Global developmental delay and intellectual disability were the most common findings, followed by seizures [5]. Observed facial features include upslanted palpebral fissures, epicanthal folds, depressed nasal bridge, low-set ears, hypertelorism, frontal bossing, and micrognathia [5].

Temtamy syndrome is considered one of the rarest genetic disorders, with around 58 cases documented up to this date [3-8]. Middle Eastern countries accounted for 54 cases of Temtamy syndrome, largely in Saudi Arabia, with 25 cases, followed by Kuwait and the United Arab Emirates with 9 cases each [3-5]. The majority of these cases involved parental consanguinity [1-5].

This case report is the first of its kind in the current literature, which details the dental management of a child diagnosed with Temtamy syndrome, facial and oral features, and difficulties faced during treatment. This case report was written following the guidelines provided by CARE (CAse REport) [9].

## Case presentation

A four-year-old male patient with his father visited our hospital complaining of dental pain. He presented with general developmental delay, intellectual disability, and generalized hypotonia. There was no previous history of convulsions. Eyesight and hearing were normal. The patient presented with a small atrial septal defect (ASD) about 5 mm with left-to-right shunt.

Up to three years of age, the child had no words in his vocabulary, and he could only eat mushed food. While growing up, his family complained of significant snoring and sleep hypopnea/apnea, which was greatly improved after adenoidectomy surgery at two years of age. Also, hypotonia was greatly improved with physiotherapy, which helped him to walk and run. Currently, his diet has improved, and he is capable of eating soft food like rice.

The whole exome sequencing report dated on the first of December 2022 identified a homozygous pathogenic variant (c.1A>G:p.Met1Val) in exon one of the *C12orf57* gene. The genetic findings are consistent with Temtamy syndrome. The patient's parents were consanguineous.

The father provided a brain MRI report that mentioned no corpus callosum abnormalities. The MRI scan was performed when the child was three of years of age at another facility. Facial dysmorphic features included frontal bossing, hypertelorism of the eyes, upslanting palpebral fissures, a depressed nasal bridge, low-set ears, and micrognathia.

Clinical findings

**Figure 1:**
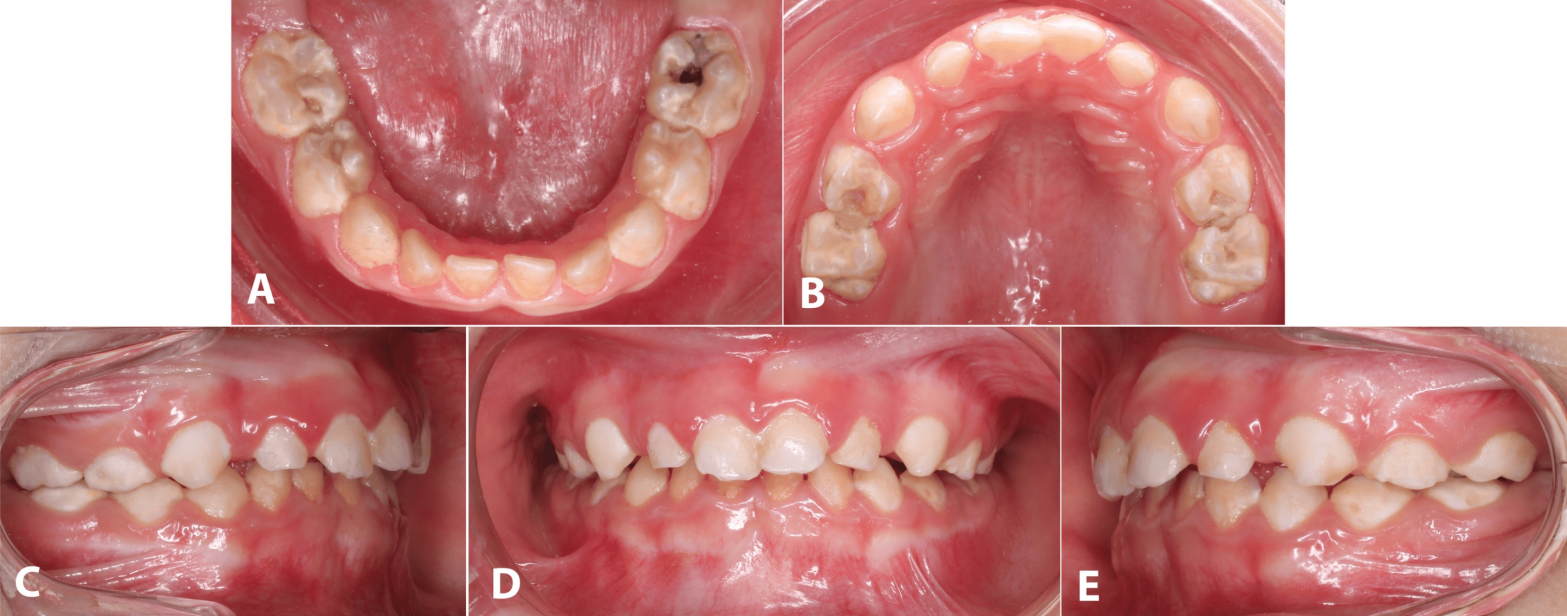
Perioperative dental photographs (A, B) Occlusal view of the teeth with deep caries. (C-E) Heavy plaque deposits with inflammed gingival tissues.

Oral examination revealed poor oral hygiene with heavy plaque on most of the teeth. Plaque induced gingivitis of the gums. The patient had occlusal caries in teeth #55, 54, 64, 65, 75, 74, 84, and 85, as well as proximal caries between teeth #82 and 83. Patient also had increased overjet of upper anterior teeth. Micrognathia with a limited mouth opening was present (Figure 1).

Radiographic assessment

**Figure 2:**
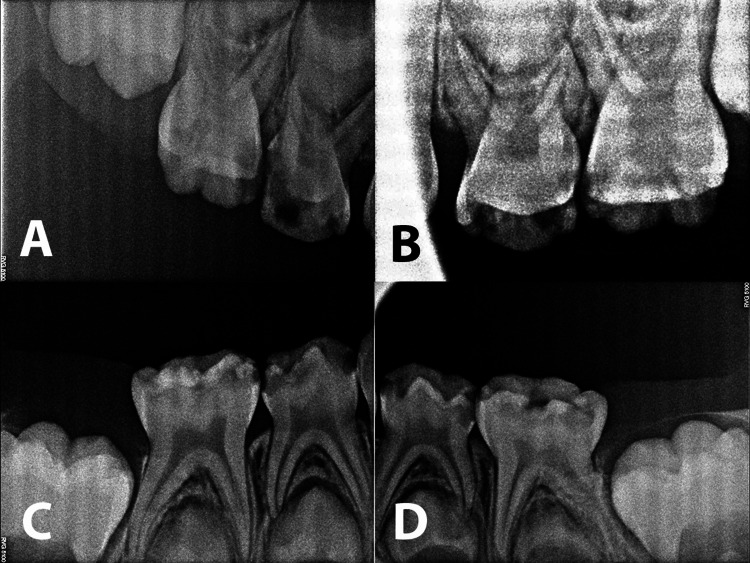
Perioperative dental radiographs of the patient (A-D) Periapical X-rays showing deep occlusal and interproximal carious lesions in the primary molars.

Radiographs were taken in the operating room after induction of anesthesia. A total of four periapical X-rays were taken, targeting teeth with deep caries. Radiographic examination revealed deep carious lesions in all primary molars nearing the pulpal tissues of the teeth (Figure 2).

Dental intervention

Behavior difficulty of the patient required dental rehabilitation under general anesthesia. Consultation with the cardiac department regarding the ASD revealed no precautions to be taken before dental intervention.

The patient was then sent to the anesthesia clinic for assessment before the operation. The patient was classified as Mallampati score II, with no difficulties in intubation anticipated. Laboratory results were within normal ranges, and the patient was accepted for dental rehabilitation under general anesthesia. It was advised that patient will need antibiotic prophylaxis before the operation. Nasal intubation was planned to increase accessibility of dental treatment. The patient was to be admitted to the day surgery unit on the day of the operation.

During dental treatment, micrognathia of the mandible led to difficulties, as the patient had a limited mouth opening, making the second primary molar hard to reach. Teeth #55 and 65 required stainless steel crowns (SSCs). Meanwhile, teeth #83 and 82 received composite fillings. Dental extractions were planned for teeth #54, 64, 75, 74, 84, and 85. Extraction was planned considering several factors, which included: deep carious lesions, pulpal involvement, high caries risk, poor oral hygiene, the patient’s medical status, and to provide a definitive treatment with the highest rate of success in the long-term.

**Figure 3:**
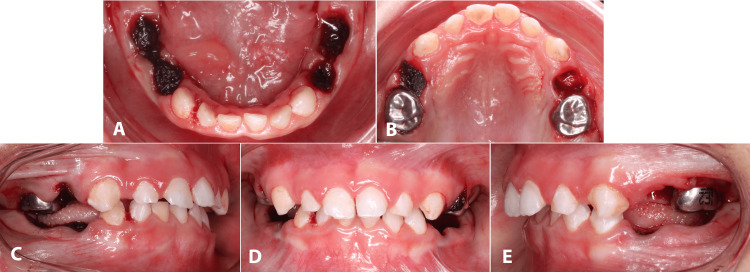
Postoperative dental treatment photographs (A, B) Extraction sites packed with an oxidized regenerated cellulose. (C-E) Lateral and frontal views of provided dental treatment.

Extraction sites were packed with an oxidized regenerated cellulose to reduce postoperative bleeding. The patient was discharged from hospital, as he was stable and passed the required criteria needed for discharge (Figure 3).

Follow-up and outcomes

A follow-up appointment was given after two weeks. The patient had no complaints, was eating well, and had good oral hygiene. Restorations and SSCs were intact. The patient was scheduled for regular follow-ups every six months.

## Discussion

The current published literature on Temtamy syndrome has no articles detailing the dental treatment aspects of such patients. To our knowledge, this is the first documented case to describe the dental features, oral findings, and clinical management of a patient with Temtamy syndrome.

The founder mutation of gene *C12orf57* is c.1A>G, p.(Met1?), accounting for 80.3% of all Temtamy syndrome cases [5]. Currently, there are more than seven gene mutations of *C12orf57* accounting for the Temtamy syndrome as expressed in the literature [1-8]. The majority of cases presented with global developmental delay [5]. Other common neurological findings reported in the literature were absence of speech, seizures, autistic behavior, and generalized hypotonia [1-8]. Cardiac anomalies included ASD (29.1%), followed by pulmonic stenosis (11.54%) and ventricular septal defect (10.5%) [5]. Brain MRI scans showed abnormalities of the corpus callosum in 63% of cases [5]. About two-thirds of Temtamy syndrome patients had dysmorphic facial features [1-8].

Treating children with special health care needs (SHCN) can be difficult and challenging. Patients with SHCN experience an elevated level of anxiety toward dental care when compared with regular patients [10]. Accurate dental diagnosis, pain management, and dental treatment prove to be complicated in such cases. Common challenges include the inability to perform clinical examination and take intraoral radiographs. Patients are unable to express their feelings, the nature of pain, and its origin. Asking parents about the dental pain, including its duration and nature, could be helpful in diagnosis and treatment planning. Oftentimes, patients have diseases that go undiagnosed, and many symptoms or features are first noticed by the dentist in the dental clinic. Many patients live in rural areas or travel a great distance to receive dental treatment, making it difficult to attend regular follow-ups or reach the hospital during emergencies. Clinicians should therefore review every case thoroughly to assess the child’s needs, parents’ expectations, and unique challenges in each case, in order to construct the proper dental treatment that best suits the child and their parents. Prevention is the gold standard in treating special needs children, which includes proper education of patients and parents about the importance of oral hygiene practices, and providing anticipatory guidance with follow-up visits [10].

Micrognathia is a common facial dysmorphic feature found in cases of Temtamy syndrome [5]. The patient’s family complained of snoring and sleeping hypopnea/apnea when he was younger; micrognathia could be the reason for these symptoms. Micrognathia is also likely the cause of the limited mouth opening. If the patient is treated under local anesthesia in the dental clinic, the limited mouth opening alone could complicate the treatment, and thus the patient would require treatment under general anesthesia to overcome such a problem.

While our case had no difficulties during nasal endotracheal intubation, micrognathia may lead to challenges during intubation of the patient, and the anesthesiologist might choose oral endotracheal intubation to overcome such problems, which further complicates the dental treatment due to limited mouth opening and the endotracheal tube position, which further restricts access to the teeth.

## Conclusions

Each case of Temtamy syndrome presents with different presentations and challenges. Pediatric dentists should expect micrognathia and a limited mouth opening when treating such cases. Dental rehabilitation under general anesthesia is the preferred way to overcome these problems. Intubation could prove difficult due to micrognathia. Cardiac anomalies are also expected with such patients; thus, a detailed medical history should be taken with proper consultations. A multidisciplinary team approach is key to successful treatment. Clinicians focus should be on prevention, anticipatory guidance, and regular follow-ups. They need to carefully assess each patient to construct the proper treatment that suits the child and the parents’ needs. More studies are needed to further understand the Temtamy syndrome, discover more of its oral features, and help fellow clinicians in managing such patients.
